# ﻿New data on the scale insect (Hemiptera, Coccomorpha) fauna of Iceland, with description of a new species

**DOI:** 10.3897/zookeys.1236.150789

**Published:** 2025-04-29

**Authors:** Kornél Gerő, Matthías Alfreðsson, Éva Szita

**Affiliations:** 1 National Laboratory for Health Security, Plant Protection Institute, Centre for Agricultural Research, HUN-REN, H-2462 Martonvásár, Brunszvik u. 2, Budapest, Hungary Centre for Agricultural Research Budapest Hungary; 2 Náttúrufræðistofnun, Natural Science Institute of Iceland, Urriðaholtsstræti 6-8, 210 Garðabær, Iceland Natural Science Institute of Iceland Garðabær Iceland

**Keywords:** Adventive species, checklist, identification key, mealybug, new species, Pseudococcidae, Sternorrhyncha, taxonomy, *Trionymus*

## Abstract

This study adds seven species to the scale insect species list of Iceland, bringing the total number of recorded species to 15. Of these, 10 species can be considered as a part of the country checklist with breeding populations in Iceland (seven species can be found in outdoor conditions and three live indoors). An additional five species were recorded on imported fruits and most probably are not established in Iceland. A new species, *Trionymusicelandensis* Gerő & Szita, **sp. nov.** (Hemiptera: Pseudococcidae) is described from outdoor habitats, and the adult female is illustrated.

## ﻿Introduction

Scale insects (Hemiptera: Sternorrhyncha: Coccomorpha) are a substantial and diverse group, with over 8400 described species of small, obligate plant parasites (García Morales et al. 2016). The insects, often measuring less than 5 mm in length, are notorious as agricultural pests, posing a significant threat to a wide range of plant species (Kosztarab and Kozár 1988; Gullan and Martin 2009). Cryptic in their habits, scale insects are adept at evading detection at plant quarantine inspection, which enhances their potential for introduction to new regions globally (Watson 2002; Mazzeo et al. 2014). Their high adaptability enables them to establish populations in diverse environments, including urban parks, agricultural plantations, and tropical greenhouses (Williams and Pellizzari 1997; Kozár 1998; Kondo and Watson 2022). Climate change, particularly increases in temperature, can facilitate the colonization and establishment of scale insects in new regions; this can have serious economic and ecological consequences (Kozár 2009; Kozár et al. 2013b; Gertsson 2023).

Iceland, a Nordic island country located on the Mid-Atlantic Ridge between North America and Europe, is the second largest island in Europe, covering an area of 103,000 km^2^ (Thordarson and Höskuldsson 2014). The shortest distances to its nearest neighbours are approximately 280 km to Greenland, 400 km to the Faroe Islands, 800 km to Scotland, and 970 km to Norway (Denk et al. 2011). The climate ranges from Arctic in the far north to subarctic and temperate along the coastlines. This environmental heterogeneity contributes to a complex mosaic of ecosystems, including moss-covered lava fields, geothermal landscapes, glacial expanses, and coastal habitats. Iceland supports a rich biodiversity, with fauna and flora exhibiting unique adaptations to the island’s specific ecological zones and variable weather conditions (Denk et al. 2011).

The scale insect fauna of Iceland is largely unexplored. The earliest mention of scale insects there dates back to 1772 (Olafsen and Povelsen 1772), when *Arctortheziacataphracta* (Olafsen, 1772) (Hemiptera: Ortheziidae) was described. For the next 160 years, this remained the only recorded scale species in Iceland (Müller 1776; Mohr 1786; Staudinger 1857; Lindroth 1928). Green (1931) later described four new species from Iceland based on specimens collected by the Swedish entomologist Carl H. Lindroth. In a comprehensive study, Ossiannilsson (1955) redescribed *Trionymusincertus* Green, 1931 (Hemiptera: Pseudococcidae), which originally was described from an immature female, reported several new locations of scale insect species, and referenced a few authors (Fristrup 1943; Gígja 1944; Björnsson 1951) who had previously investigated Iceland´s scale insect fauna. Two greenhouse-inhabiting species were also reported by Gígja (1944) and Ossiannilsson (1955). Lindroth (1965) published the most recent scale insect data for Iceland, *Chionaspissalicis* (Linnaeus, 1758), an introduced outdoor species.

Our knowledge of cold-tolerant scale insect species is rather poor; to date, eight species of scale insects have been recorded from Iceland. The present work provides new data on the scale insect fauna of Iceland, including a description of a new *Trionymus* species adapted to the harsh outdoor conditions, a country checklist, and a list of adventive scale insect species in Iceland. Furthermore, an identification key is provided to the *Trionymus* species of Iceland.

## ﻿Material and methods

During a brief survey in Iceland, 31 scale insect samples were collected between 15 and 30 September 2022 by the first author. Among these, 21 samples were collected from infested tropical and subtropical fruits in supermarkets and grocery stores in the towns of Reykjavík, Selfoss, and Vík í Mýrdal. Additionally, eight samples were collected from a greenhouse in the Reykjavík Botanical Garden. Moreover, ten soil samples were collected outdoors in Vík í Mýrdal, but only two of them provided scale insect specimens. The soil samples were processed in a Berlese funnel, which is a valuable tool for extracting arthropods and other small invertebrates from soil, leaf litter, and other organic matter (Southwood and Henderson 2000); it consists of a metal or plastic funnel, a heat and light source, and a collection container. The sample is placed in the funnel, and the heat source is positioned above. The heat creates temperature and humidity gradients within the funnel, with the warmest and driest conditions at the top and the coolest and moistest at the bottom. As the sample heats up, motile organisms move away from the heat and towards the cooler bottom of the funnel. They eventually fall into the collection container below, where they can be collected and identified (Southwood and Henderson 2000; Kaydan et al. 2016). All scale insects samples were stored in 96% ethyl alcohol before transport to the laboratory at HUN-REN Centre for Agricultural Research, Plant Protection Institute (PPI) in Hungary.

Specimens were prepared for light microscopy following the slide-mounting protocol described by Kosztarab and Kozár (1988). The slide-mounted specimens were examined using a phase-contrast light microscope (Olympus BX41) and identified using the published keys available in Borchsenius (1949), Tang (1992), Williams and Granara de Willink (1992), Danzig (1993), Hodgson (1994), Williams (2004), Miller and Davidson (2005), and Danzig and Gavrilov-Zimin (2014, 2015).

The type specimens of the new species are deposited in the Natural Science Institute of Iceland (NSII). For the holotype of the new species, the data on the label is listed with “/” indicating each line break. Voucher slides of the other species collected were deposited in the collections at PPI and NSII.

## ﻿Results

A total of 31 scale insect samples were collected during the survey, representing seven species belonging to three families (Tables 1, 2).

**Table 1.** Country checklist of scale insect fauna (Hemiptera: Coccomorpha) of Iceland, with comments on current collecting records and information on their occurrence in Iceland.

Taxon	Comments	Occurrence in Iceland
**Diaspididae** (2 genera)		
*Chionaspissalicis* (Linnaeus, 1758)	First recorded by Lindroth (1965)	Outdoors
*Pinnaspisaspidistrae* (Signoret, 1869)	Previously recorded as *Hemichionaspisaspidistrae*, first recorded by Ossiannilsson (1955).	Indoors
**Coccidae** (1 genus)		
*Coccushesperidum* Linnaeus, 1758	**New country record for Iceland.** Reykjavík: From the greenhouse of the Reykjavik Botanical Garden on *CalycanthusAphrodite*, *Clematis‘Jackmanii’*, *Libertiagrandiflora*, *Magnoliagrandiflora*, *Sarcococcaconfusa*, *Wisteriafloribunda*.	Indoors
**Pseudococcidae** (3 genera)		
*Trionymusincertus* Green, 1931	First recorded by Green (1931).	Outdoors
*Trionymusicelandensis* Gerő & Szita, sp. nov.	**New country record for Iceland. New to science.** Vík í Mýrdal, samples from Berlese funnel: *Festucavivipara*, *Poapratensis.*	Outdoors
*Trionymusthulensis* Green, 1931	First recorded by Green (1931).	Outdoors
*Pelionellabalteata* (Green, 1928)	Previously recorded as *Phenacoccusvenustus*, first published by Green (1931).	Outdoors
*Pseudococcusmaritimus* (Ehrhorn, 1900)	First recorded by Gígja (1944).	Indoors
**Ortheziidae** (1 genus)		
*Arctortheziacataphracta* (Olafsen, 1772)	First recorded by Olafsen and Povelsen (1772).	Outdoors
**Acanthococcidae** (1 genus)		
*Anophococcusgranulatus* (Green, 1931)	First recorded by Green (1931).	Outdoors

**Table 2.** List of adventive scale insect species found on imported fruits in Iceland.

Taxon	Comments
**Diaspididae** (5 genera)	
*Aonidiellaaurantii* (Maskell, 1879)	**New record for Iceland.** Reykjavík, Bónus supermarket, on *Citrusaurantiifolia* imported from Brazil. Vík í Mýrdal, Krónan supermarket, on *Citrussinensis* and *Citrus×clementina* imported from Spain.
*Lepidosaphesbeckii* (Newman, 1869)	**New record for Iceland.** Selfoss, Bónus supermarket, on *Citrussinensis* imported from Spain. Vík í Mýrdal, Krónan supermarket on *Citrussinensis* imported from Spain.
*Lepidosaphesgloverii* (Packard, 1869)	**New record for Iceland.** Vík í Mýrdal, Krónan supermarket on *Citrussinensis* imported from Spain.
*Parlatoriapergandii* Comstock, 1881	**New record for Iceland.** Selfoss, Krónan supermarket on *Citrussinensis* imported from Spain.
*Pseudaulacaspispentagona* (Targioni Tozzetti, 1886)	**New record for Iceland.** Vík í Mýrdal, Krónan supermarket on *Actinidiadeliciosa*.

A single species of soft scale insect (Coccidae), namely *Coccushesperidum* Linnaeus, 1758, was collected from five different host-plant species from a greenhouse in Reykjavik Botanical Garden, and proved to be new to the Icelandic fauna.

Five armored scale insect species (Diaspididae) proved to be new records to Iceland. All the diaspidid specimens were collected from imported fruits, namely *Aonidiellaaurantii* (Maskell, 1879), *Lepidosaphesgloverii* (Packard, 1869), *Lepidosaphesbeckii* (Newman, 1869), *Parlatoriapergandii* Comstock, 1881, and *Pseudaulacaspispentagona* (Targioni Tozzetti, 1886). These species are most probably not established and not breeding in Iceland, thus can’t be considered as a part of the country’s fauna, but as recorded in Iceland, belong to the scale insect species list of Iceland (Table 2).

An analysis of ten soil samples utilizing Berlese funnels, resulted in the detection of scale insects in two instances. This study led to the identification and description of a previously unknown species within the mealybug genus *Trionymus* (Pseudococcidae). A generic diagnosis of *Trionymus* can be found in Danzig and Gavrilov-Zimin (2015).

### ﻿ Trionymus icelandensis

Taxon classificationAnimaliaHemipteraPseudococcidae

Gerő & Szita sp. nov.

EEC99F95-5B7B-5DCE-B4AF-55258407F18A

https://zoobank.org/9B389D56-DBFB-44D6-9EF3-9B1B466E1260

Fig. 1

#### Material examined.

***Holotype*** • 1 adult ♀ mounted singly on a slide; left label: NSII 113860 / PPI 13482 (work) / ICELAND / Vík í Mýrdal / 63°25'13"N, 18°59'58"W / 15 Sep. 2022 / *Festucavivipara*; right label: *Trionymus* / *icelandensis* / Gerő & Szita / 1 ♀, holotype / Leg. K. Gerő / Det. É. Szita. ***Paratype*** • 1 adult ♀ mounted singly on a slide; ICELAND, Vík í Mýrdal; 63°25'12"N, 19°00'06"W; 16 Sep. 2022; K. Gerő leg.; *Poapratensis*; NSII code: 113859; PPI work code: 13481. (Both holotype and paratype are deposited in NSII)

#### Description.

***Unmounted adult female*.** Body elongate oval, light yellow, covered with fine powdery wax.

***Slide-mounted adult female*.** Body elongate oval, 1.68–1.69 mm long, 0.83–0.86 mm wide. Eyes marginal, each 24–26 μm wide. Antennae each 6 segmented, 258–264 μm long in total. Length of antennal segments: 1^st^ 36–43.2 μm, 2^nd^ 31.2–36 μm, 3^rd^ 38.4–43.2 μm, 4^th^ 19.2 μm, 5^th^ 24–28.8 μm, 6^th^ 72.8–79.2 μm, segments nearly parallel sided. Apical segment with 1 apical seta, 32–34 μm long; with 4 subapical setae, each 30–32 μm long, and with 5 fleshy setae, each 32–35 μm long. 5^th^ segment with 1 fleshy seta, 32 μm long. Other setae throughout the segments hairlike, slightly curved with fine tip, 25–40 μm long. Clypeolabral shield not visible. Labium 3 segmented, 80–91 μm long, 81–82 μm wide, 5 or 6 setae each 12–20 μm long. Anterior spiracles each 36–38 μm long, and about 14 μm wide across atrium; posterior spiracles each 40–42 μm long, and about 15 μm wide across atrium. Legs well developed; hind leg without translucent pores, segment lengths (in μm): coxa 68–75; trochanter + femur 165–168; tibia + tarsus 207–210; claw 22–23. Ratio of lengths of tibia + tarsus to trochanter + femur 1: 1.23–1.27; ratio of lengths of tibia to tarsus 1: 1.14–1.27; ratio of lengths of hind trochanter + femur to greatest width of femur 1: 2.37–2.65. Tarsal digitules hairlike, each 27–30 μm long. Claw digitules capitate, 24–25 μm long. Both pairs of ostioles present, lips not sclerotized; anterior ostioles each with a total for both lips of 5–8 trilocular pores and no setae; posterior ostioles each with a total for both lips of 7 or 8 trilocular pores and no setae. Anal ring 74–75 μm wide, with two complete rows of pores, the outer row with spiculae, ring bearing 6 setae, each seta 110–130 μm long.

**Figure 1.**
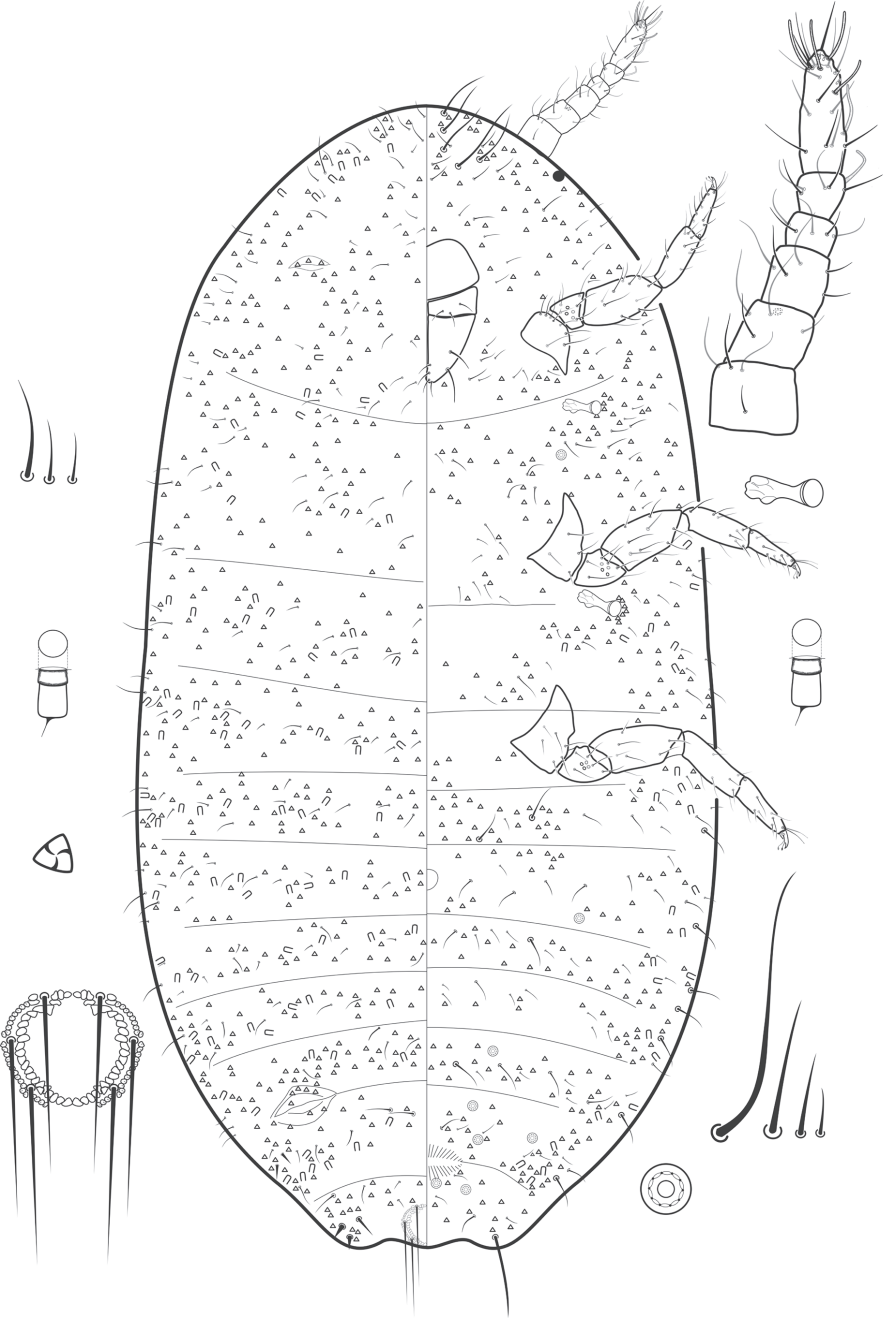
*Trionymusicelandensis* Gerő & Szita, sp. nov., holotype female. On antenna and legs, ventral setae are coloured grey, and dorsal setae are black.

***Dorsum*.** Derm membranous; with two pairs of cerarii on last abdominal segments. Setae flagellate, slightly curved, of 3 sizes: small setae each 12–15 μm long; mid-sized setae each 19–23 μm long; and longest setae each 27–30 μm long. Longest setae distributed mainly marginally, others scattered throughout. Trilocular pores numerous throughout, each about 3.2 μm in diameter. Oral collar tubular ducts of one size, outer ductule 4 μm wide, 6.5 μm long. Multilocular disc-pores absent.

***Venter*.** Derm membranous; one small circulus, present on middle of abdominal segment III, 24 μm long and 26.4 μm wide. Apical seta on each anal lobe 130–132 μm long. Body setae flagellate, slightly curved, in 4 sizes: shortest setae each 11–14 μm long, present throughout; middle-sized setae each 25– 26 μm long, present throughout; second longest setae each 40–43 μm long, situated on margins of abdomen and a few present in medial zone of abdomen; and longest setae each 66–80 μm long, situated medially on head. Trilocular pores numerous, each about 3.2 μm in diameter. Oral collar tubular ducts of one size, same as on dorsum. Multi­locular disc-pores, each about 8 μm in diameter with 10 loculi, numbering 2–8, present on abdominal segments III–VIII and occasionally also on mesothorax.

#### Diagnosis.

*Trionymusicelandensis* Gerő & Szita, sp. nov. can be recognised by possessing the following combination of features: (i) antennae each six segmented; (ii) eyes present; (iii) legs well developed, without translucent pores; (iv) one small circulus; (v) oral collar tubular ducts of one size present on both surfaces; (vi) multilocular disc-pores few, present on venter only, on abdominal segments III–VIII and occasionally on thoracic segment II; and (vii) body setae flagellate, in 3 sizes on dorsum, and in 4 sizes on venter, longest ones on venter of head.

#### Comments.

*Trionymusicelandensis* is similar to three other species of *Trionymus*. It resembles *T.artemisiarum* (Borchsenius, 1949) in having two pairs of cerarii, lacking multilocular pores on dorsum, and in having 6-segmented antennae; however, it differs as follows (character states of *T.artemisiarum* are given in brackets): (i) having one circulus (circulus absent); (ii) oral collar tubular ducts of one size (two sizes); and (iii) hind coxa without translucent pores (translucent pores present).

*Trionymusicelandensis* resembles *T.massiliensis* (Goux, 1941) in lacking multilocular pores on dorsum; however, it differs as follows (character states of *T.massiliensis* are given in brackets): (i) having two pairs of cerarii (one pair); (ii) one circulus (circulus absent); (iii) oral collar tubular ducts of one size (two sizes); (iv) hind coxa without translucent pores (translucent pores present); and (v) antennae each with six segments (seven segments).

*Trionymusicelandensis* resembles *T.thulensis* Green, 1931 in having two pairs of cerarii, one circulus, and in lacking multilocular pores on dorsum; however, it differs as follows (character states of *T.thulensis* are given in brackets): (i) having oral collar tubular ducts of one size (two sizes); (ii) hind coxa without translucent pores (translucent pores present); and (iii) antennae each with six segments (seven or eight segments).

#### Etymology.

The species is named in homage to the country of Iceland; the epithet is formed by the combination of the island name, Iceland, with the Latin suffix “-*ensis*”, meaning “originating from”.

#### Distribution.

Iceland.

#### Host plants.

Poaceae: *Festucavivipara*, *Poapratensis*.

##### ﻿Scale insect species list for Iceland

With this study, the total number of scale insect species with breeding populations recorded in Iceland was increased to ten species (Table 1). Of these, seven species can be found in outdoor conditions, three are indoor species. Five species were found exclusively on imported fruits and are not considered to be established in Iceland (Table 2).

## ﻿Discussion

Ossiannilsson (1955) considered four species to be endemic to Iceland, namely *Anophococcusgranulatus* (Green, 1931) (Hemiptera: Acanthococcidae), *Pelionellabalteata* (Green, 1928), *Trionymusincertus* Green, 1931 and *T.thulensis* Green, 1931 (Hemiptera: Pseudococcidae). However, since the study by Ossiannilsson (1955), three of these species have also been found in other countries, meaning their endemic status is no longer confirmed. *Anophococcusgranulatus* has been recorded in France (Foldi 2001) and in Hungary (Kozár et al. 2013a). *Pelionellabalteata* was described under the name *P.venustus* by Green from Iceland in 1931, a synonymy discovered by Danzig (2001). *Pelionellabalteata* seems to have a Holarctic distribution, as it has been found in 12 countries (García Morales et al. 2016). *Trionymusthulensis* has been reported from eight Palaearctic countries (García Morales et al. 2016) since its description from Iceland.

The redescription of *Trionymusincertus* Green, 1931 by Ossiannilsson (1955) was considered insufficient by Danzig and Gavrilov-Zimin (2015); but at the same time, it is accepted by ScaleNet (García Morales et al. 2016). In our opinion, the redescription made by Ossiannilson can be used effectively in distinguishing the currently known *Trionymus* species in Iceland, although a redescription and redrawing of this species would be necessary to fulfil the requirements of modern taxonomy.

### ﻿Identification key to *Trionymus* species found in Iceland

**Table:** 

1	With 1 ventral circulus	**2**
–	With 2 ventral circuli	***Trionymusincertus* Green, 1931**
2	Abdominal segments VI–VIII with more than 15 ventral multilocular disc pores; antennae each eight segmented	***Trionymusthulensis* Green, 1931**
–	Abdominal segments VI–VIII with fewer than 15 ventral multilocular disc pores; antennae each six segmented	***Trionymusicelandensis* Gerő & Szita, sp. nov.**

To date, only eight species have been reported from Iceland, but with this study our knowledge of scale insects in Iceland has been improved significantly. The total number of scale insect species recorded on the territory of Iceland has been increased to 15. Of these, ten species can be considered as a part of the country checklist, with breeding populations in Iceland. Furthermore five adventive species were also registered from imported fruits, which most probably are not established in Iceland.

For comparison, in nearby Greenland, nine species have been recorded (Morrison 1925; García Morales et al. 2016), and only one from Faeroe Islands (Annandale 1904). Among the subarctic countries in Europe, the scale insect fauna of Sweden is the most explored, with 108 recorded species (Gertsson 2001; García Morales et al. 2016), while Norway has 23, Scotland 32, and Finland 25 species (García Morales et al. 2016). These data suggest that it is worthwhile to make efforts to explore the scale insect fauna of Iceland in more detail in the future. The study of adventive species is also worth paying attention to, as they might become potential invaders indoors or outdoors, depending on the species.

## Supplementary Material

XML Treatment for Trionymus icelandensis

## ﻿Additional information

### Author ORCIDs

Kornél Gerő https://orcid.org/0000-0002-0366-0050

Matthías Alfreðsson https://orcid.org/0009-0006-5418-891X

Éva Szita https://orcid.org/0000-0001-6335-5296

### Data availability

All of the data that support the findings of this study are available in the main text.

## References

[B1] Annandale N (1904) Contributions to the terrestrial zoology of the Faroes. Proceedings of the Royal Society of Edinburgh (Phys) 15: 153–160.

[B2] Björnsson H (1951) Gróður og dýralíf í Esjufjöllum. Náttúrufræðingurinn 21: 109–112.

[B3] Borchsenius NS (1949) Fauna of USSR (Homoptera, Pseudococcidae). [in Russian]. Akademii Nauk Zoologicheskogo Instituta Leningrad (n.s. 38), Leningrad, 383 pp.

[B4] Danzig EM (1993) [Fauna of Russia and neighbouring countries. Rhynchota, Volume X: suborder scale insects (Coccinea): families Phoenicococcidae and Diaspididae.]. ‘Nauka’ Publishing House, St. Petersburg, 452 pp.

[B5] Danzig EM (2001) Mealybugs of the genera *Peliococcus* and *Peliococcopsis* from Russia and neighbouring countries (Homoptera: Coccinea: Pseudococcidae). Zoosystematica Rossica 9: 123–154.

[B6] Danzig EM Gavrilov-Zimin IA (2014) Palaearctic mealybugs (Homoptera: Coccinea: Pseudococcidae), Part 1: Subfamily Phenacoccinae Russian Academy of Sciences, Zoological Institute, St. Petersburg, 678 pp.

[B7] Danzig EM Gavrilov-Zimin IA (2015) Palaearctic Mealybugs (Homoptera: Coccinea: Pseudococcidae) Part 2. Fauna of Russia and Neighbouring Countries, New series, No. 149. Zoological Institute, Russian Academy of Sciences, St. Petersburg, 619 pp.

[B8] Denk T Grimsson F Zetter R Símonarson LA (2011) Late Cainozoic Floras of Iceland. 15 Million Years of Vegetation and Climate History in the Northern North Atlantic. Springer, Dordrecht, 845 pp. 10.1007/978-94-007-0372-8

[B9] Foldi I (2001) Liste des cochenilles de France (Hemiptera, Coccoidea). Bulletin de la Société entomologique de France 106: 303–308. 10.3406/bsef.2001.16768

[B10] Fristrup B (1943) Contributions to the Fauna and Zoogeography of Northwest Iceland. Entomologiske Meddelelser 23: 148–173.

[B11] García Morales M Denno BD Miller DR Miller GL Ben-Dov Y Hardy NB (2016) ScaleNet: a literature-based model of scale insect biology and systematics. Database. [Date accessed: 26.03.2025] 10.1093/database/bav118 PMC4747323 26861659

[B12] Gertsson CA (2001) An annotated checklist of the scale insects (Homoptera: Coccoidea) of Sweden. [Förtechnung över Sveriges sköldlöss. In English; summary in Swedish]. Entomologisk Tidskrift Stockholm 122: 123–130.

[B13] Gertsson CA (2023) Första fyndet av den invasiva sköldlusen *Pseudaulacaspispentagona* (Tagioni Tozzetti, 1886) påfriland i Sverige (Hemiptera, Coccomorpha, Diaspididae). [The first outdoor record of the invasive scale insect *Pseudaulacaspispentagona* (Tagioni Tozzetti, 1886) in Sweden (Hemiptera, Coccomorpha, Diaspididae). In Swedish]. FaZett 36: 20–26.

[B14] Gígja G (1944) Meindýr í húsum og gródri og varnir gegn beim. Jens Guđbjörnsson, Reykjavík, 235 pp.

[B15] Green EE (1931) Notes on some Coccidae from Iceland. Entomologisk Tidskrift 52: 263–269.

[B16] Gullan PJ Martin JH (2009) Sternorrhyncha (jumping plant-lice, whiteflies, aphids, and scale insects). In: Resh VH Cardé RT (Eds) Encyclopedia of Insects 2nd (Ed.) Elsevier, San Diego, 957–967. 10.1016/B978-0-12-374144-8.00253-8

[B17] Hodgson CJ (1994) The scale insect family Coccidae: an identification manual to genera. CAB International, Wallingford, 639 pp.

[B18] Kaydan MB Konczné Benedicy Z Kiss B Szita É (2016) A survey of scale insects in soil samples from Europe (Hemiptera, Coccomorpha). ZooKeys 565: 1–28. 10.3897/zookeys.565.6877 PMC4820095 27081335

[B19] Kondo T Watson GW (Eds) (2022) Encyclopedia of scale insect pests. CAB International, Wallingford, UK, 640 pp. 10.1079/9781800620643.0000

[B20] Kosztarab M Kozár F (1988) Scale Insects of Central Europe. Akadémiai Kiadó, Budapest, 456 pp. 10.1007/978-94-009-4045-1

[B21] Kozár F (1998) Éghajlatváltozás és a rovarvilág. Magyar Tudomány 9: 1069–1076.

[B22] Kozár F (2009) Pajzstetű (Hemiptera: Coccoidea) fajok és a klímaváltozás: vizsgálatok Magyarországi autópályákon [Scale insect species (Hemiptera, Coccoidea) and climate change studies on Hungarian highways]. Növényvédelem 45: 577–588.

[B23] Kozár F Konczné Benedicty Z Fetykó K Kiss B Szita É (2013a) An annotated update of the scale insect checklist of Hungary (Hemiptera, Coccoidea). ZooKeys 309: 49–66. 10.3897/zookeys.309.5318 PMC3689126 23794928

[B24] Kozár F Szita É Fetykó K Neidert D Konczné Benedicty Z Kiss B (2013b) Pajzstetvek, sztrádák, klíma. MTA ATK Növényvédelmi Intézet, Budapest, 216 pp.

[B25] Lindroth CH (1928) Zur Land-Evertebratenfauna Islands. Göteborgs Kungl. vetenskaps- och vitterhets- samhälles handlingar, 52 pp.

[B26] Lindroth CH (1965) Skaftafell, Iceland, a living glacial refugium. Oikos Suppl. 6: 142 pp.

[B27] Mazzeo G Longo S Pellizzari G Porcelli F Suma P Russo A (2014) Exotic scale insects (Coccoidea) on ornamental plants in Italy: a never-ending story. Acta Zoologica Bulgarica suppl. 6: 55–61.

[B28] Miller DR Davidson JA (2005) Armored Scale Insect Pests of Trees and Shrubs. Cornell University Press, Ithaca, NY, 442 pp.

[B29] Mohr N (1786) Forsøg til en Islandsk Nuturhistoric. Trykt hos C. F. Holm, 413 pp.

[B30] Morrison H (1925) Classification of scale insects of the subfamily Ortheziinae. Journal of Agricultural Research 30: 97–154.

[B31] Müller OF (1776) Zoologiae Danicae prodromus, seu, Animalium Daniae et Norvegiae indigenarum characteres, nomina, et synonyma imprimis popularium. Typis Hallageriis, Havniae, 282 pp. 10.5962/bhl.title.13268

[B32] Olafsen E Povelsen B (1772) Reise igiennem Island. Soroe, 1042 pp.

[B33] Ossiannilsson F (1955) Hemiptera 3. Coccina, Aleyrodina and Psyllina The Zoology of Iceland III: 1–12.

[B34] Southwood TRE Henderson PA (2000) Ecological methods. 3^rd^ edn. Blackwell Science, Oxford, 593 pp.

[B35] Staudinger O (1857) Reise nach Island zu entomologischen Zwecken unternommen. Entomologische Zeitung 18: 209–289.

[B36] Tang FT (1992) The Pseudococcidae of China. Shanxi Agricultural University, Taigu, Shanxi, China, 768 pp.

[B37] Thordarson T Höskuldsson Á (2014) Iceland. Dunedin, 256 pp.

[B38] Watson GW (2002) Arthropods of Economic Importance: Diaspididae of the World. World Biodiversity Database. ETI Information Services (Expert Center for Taxonomic Identification), Amsterdam, Netherlands. https://diaspididae.linnaeus.naturalis.nl/linnaeus_ng/app/views/introduction/topic.php?id=3377&epi=155

[B39] Williams DJ (2004) Mealybugs of southern Asia. The Natural History Museum. Kuala Lumpur: Southdene SDN. BHD, 896 pp.

[B40] Williams DJ Granara de Willink MC (1992) Mealybugs of Central and South America. CAB International, Wallingford, 635 pp.

[B41] Williams DJ Pellizzari G (1997) Two species of mealybugs (HomopteraPseudococcidae) on the roots of Aloaceae in greenhouses in England and Italy. Bollettino di Zoologia Agraria e di Bachicoltura (Milano) Ser II 29: 157–166.

